# Reperfused hemorrhagic myocardial infarction in rats

**DOI:** 10.1371/journal.pone.0243207

**Published:** 2020-12-02

**Authors:** Anand R. Nair, Eric A. Johnson, Hsin-Jung Yang, Ivan Cokic, Joseph Francis, Rohan Dharmakumar

**Affiliations:** 1 Cedars-Sinai Medical Center, Department of Biomedical Sciences, Biomedical Imaging Research Institute, Los Angeles, CA, United States of America; 2 Department of Bioengineering, University of California, Los Angeles, CA, United States of America; 3 Division of Cardiology, Department of Medicine, University of California, Los Angeles, CA, United States of America; 4 Department of Comparative Biomedical Sciences, School of Veterinary Medicine, Louisiana State University, Baton Rouge, LA, United States of America; Virginia Commonwealth University, UNITED STATES

## Abstract

**Background:**

Intramyocardial hemorrhage following reperfusion is strongly associated with major adverse cardiovascular events in myocardial infarction (MI) patients; yet the mechanisms contributing to these outcomes are not well understood. Large animal models have been used to investigate intramyocardial hemorrhage, but they are exorbitantly expensive and difficult to use for mechanistic studies. In contrast, rat models are widely used to investigate mechanistic aspects of cardiovascular physiology, but a rat model that consistently recapitulates the characteristics of an hemorrhagic MI does not exist. To bridge this gap, we investigated the physiological conditions of MI that would create intramyocardial hemorrhage in rats so that a reliable model of hemorrhagic MI would become available for basic research.

**Methods & results:**

Sprague-Dawley rats underwent either a 90-minute (90-min) ischemia and then reperfusion (I/R) (n = 22) or 30-minute (30-min) I/R (n = 18) of the left anterior descending coronary artery. Sham rats (n = 12) were used as controls. 90-min I/R consistently yielded hemorrhagic MI, while 30-min I/R consistently yielded non-hemorrhagic MI. Twenty-four hours post-reperfusion, ex-vivo late-gadolinium-enhancement (LGE) and T2* cardiac MRI performed on excised hearts from 90-min I/R rats revealed colocalization of iron deposits within the scarred tissue; however, in 30-min I/R rats scar was evident on LGE but no evidence of iron was found on T2* CMR. Histological studies verified tissue damage (H&E) detected on LGE and the presence of iron (Perl’s stain) observed on T2*-CMR. At week 4 post-reperfusion, gene and protein expression of proinflammatory markers (TNF-α, IL-1β and MMP-9) were increased in the 90-min I/R group when compared to 30-min I/R groups. Further, transmission electron microscopy performed on 90-min I/R myocardium that were positive for iron on T2* CMR and Perl’s stain showed accumulation of granular iron particles within the phagosomes.

**Conclusion:**

Ischemic time prior to reperfusion is a critical factor in determining whether a MI is hemorrhagic or non-hemorrhagic in rats. Specifically, a period of 90-min of ischemia prior to reperfusion can produce rat models of hemorrhagic MI, while 30-minutes of ischemia prior to reperfusion can ensure that the MIs are non-hemorrhagic. Hemorrhagic MIs in rats result in marked increase in iron deposition, proinflammatory burden and adverse left—ventricular remodeling compared to rats with non-hemorrhagic MIs.

## Introduction

Myocardial infarction (MI) typically results from sudden blockage of the epicardial coronary artery leading to acute myocardial ischemia, which drives cardiomyocyte necrosis with potential compromise of microvascular bed supplied by the culprit coronary artery [1]. The recommended intervention for acute MI is mechanical revascularization by percutaneous coronary intervention (PCI), which is proven to be beneficial in reducing mortality [2, 3]. Although PCI can successfully restore epicardial coronary patency, cardiac function is impaired or is progressively impaired in nearly 50% of these patients, despite revascularization/reperfusion [4]. Failure to reestablish microvascular blood flow, despite revascularization of epicardial coronary artery, known as the ‘no-reflow’ phenomenon, limits the effectiveness of the currently existing early interventional strategies in MI patients. A frequent complication associated with microvascular injury in reperfused MI is intramyocardial hemorrhage, which leads to the extravasation of red blood cells into the interstitial space [5]. Notably, the occurrence of hemorrhage within MI has been associated with the greatest risk of major adverse cardiovascular events, such as chronic heart failure and sudden cardiac death [6, 7]. However, how hemorrhage drives adverse events in the heart in the time after a reperfused myocardial infarction is unclear.

Given that large animal models have comparative cardiac anatomy to humans, prior studies investigating hemorrhage in the setting of reperfused MI have developed and employed large animal models (canine and porcine) to evaluate the effects of reperfusion hemorrhage under controlled conditions [8–11]. While these models have paralleled the clinical sequalae of hemorrhagic MI [12] and helped gather additional insight, they also pose significant practical limitations: (a) exorbitant financial burden limiting the breadth of studies, which require the assessment of time-dependent tissue-specific features from serial sacrifice of animals for tissue analysis; and (b) unparalleled difficulty in animal handling (surgery, care, housing). These challenges limit the basic science investigators to study the mechanistic aspects contributing to differential outcomes in hemorrhagic vs. non-hemorrhagic MIs.

While small animal models are widely used to investigate mechanistic aspects of cardiovascular physiology and pathophysiology, at present there are no small animal models of MI which have explored the physiological conditions driving intramyocardial hemorrhage. Specifically, the murine model provides a useful basis on which to study the differing types of tissue injury associated with MI, while keeping experimental costs low. Further, the relative lack of collateral circulation in the rat heart makes it a preferred species for studying MI [13]. Mouse models are an alternative, but it is challenging to create consistent MIs in mice; and their smaller size relative to rats pose additional difficulties for imaging studies (higher heart rates, much smaller size, etc.) [14]. To address these limitations, we investigated the specific conditions which could enable creation of a murine (rat) model of hemorrhagic MI.

We hypothesized that hemorrhagic MIs are more likely to occur when the ischemic period preceding reperfusion is extended beyond the commonly used 30–45 min ischemic period in I/R studies in the rats [15, 16]. We tested whether 1) the duration of myocardial ischemia induced via ligation of left anterior descending coronary artery (LAD) followed by reperfusion determines the type of MI (hemorrhagic vs. non-hemorrhagic MI)—early reperfusion (30-min I/R) yields non-hemorrhagic MI, while late reperfusion (90-min I/R) results in higher incidence of hemorrhagic MI, 2) hemorrhagic MI results in iron depositions in the rat myocardium, which is consistent with previous clinical and experimental reports, 3) chronic proinflammatory burden is elevated in rats with hemorrhagic MI when compared to non-hemorrhagic counterparts, 4) ultrastructural features of chronic hemorrhagic MI territories show myocardial tissue infiltration by fibroblasts, phagocytes and iron composites within phagosomes, and 5) rats with hemorrhagic MIs are associated with significantly worse left-ventricular remodeling compared to non-hemorrhagic MIs.

## Methods

### Animals

Eight-week old male Sprague-Dawley rats were used in this study. Animals were housed in a temperature-controlled (23±2°C) and light-cycled (12:12 hour light-dark cycle) room with standard chow and water provided *ad libitum*. Care of the rats met the standards set forth by the National Institutes of Health (NIH) guidelines for the care and use of experimental animals. All procedures were approved by the Louisiana State University Institutional Animal Care and Use Committee.

### Surgical procedures

Rats were anesthetized with 2% v/v isoflurane in oxygen and were placed on a heating pad in supine position and the trachea was intubated with a 24-gauge blunt-ended catheter. Anesthesia was maintained by supplementing oxygen and isoflurane using a rodent ventilator (Harvard Apparatus, Inc., MA). Left lateral thoracotomy was performed by blunt incision in the fourth intercostal space, prior to placing PE10 tubing (Fisher Scientific, USA) over the coronary artery to ligate the left-anterior descending coronary artery (LAD). Induction of ischemia was confirmed visually in the tissue supplied by LAD territory. Following the ischemic period, the PE10 tubing was removed to re-establish blood flow in the LAD. Following reperfusion, the chest was closed, and the animal was allowed to recover. Throughout the study, a successful MI was confirmed by echocardiography performed immediately after the surgery. Sham animals were used as controls and underwent the surgical procedure except ligation of the LAD. No animals died during the study or were excluded from the study.

### Experimental protocol

Two ischemia-reperfusion protocols (I/R) were used: a 30-min I/R “early reperfusion” and a 90-min I/R “late reperfusion”. 22 animals were placed in the late reperfusion group and 18 animals in the early reperfusion group (for a total N = 40). Sham rats were used as controls (n = 12). Seven animals from each group (sham, 30-min I/R and 90-min I/R) were used for chronic studies (assessment of cardiac function using echocardiography, proinflammatory cytokine expression analyses) and euthanized only after 4 weeks of I/R protocol. Rats were randomly assigned to sham, 30-min I/R or 90-min I/R groups. Sample sizes were determined by power calculation based on a pilot study. The ischemic period was initiated by ligating the LAD between the pulmonary outflow tract and the left atrium for the prescribed amount of time, followed by reperfusion of the artery. Twenty-four hours post reperfusion, a subset of rats was injected with Gd-DTPA (MRI contrast agent, i.v. tail vein) 10 minutes prior to euthanasia. Subsequently, the hearts were excised and late-gadolinium-enhancement (LGE, for detection of infarction) and T2* (for detection of hemorrhage/iron) cardiac MRI were performed (details below). Operator performing CMR analysis was blinded to the group of the rat. Hematoxylin and Eosin (H&E) staining was performed to verify tissue damage and extravasation of red blood cells; and Perl’s staining was performed to verify the presence of iron as seen on T2*-weighted images.

### Echocardiography

Deterioration of cardiac function after ischemia-reperfusion was evaluated with echocardiography under anesthesia (1.5% isoflurane/oxygen) in a cohort of rats from early- and late-reperfusion groups 4 week post-reperfusion, using Toshiba Aplio SSH770 system (Toshiba Medical Systems, Tustin, CA) fitted with a PST 65A sector scanner (8-MHz probe with capacity to generate two-dimensional images at 300 to 500 frames/s). Briefly, short-axis M-mode echocardiography was performed to measure the following parameters: Intraventricular septal diameter end diastole (IVSd) and end systole (IVSs), left ventricular (LV) internal diameter at diastole and systole (LVIDd and LVIDs respectively), LV end-systolic posterior wall thickness (PWS), LV end-diastolic posterior wall thickness (PWD) and LV ejection fraction (EF). Fractional shortening (%FS) was calculated using the equation (FS = [(LVIDd − LVIDs)/LVIDd] × 100).

### Euthanasia

At the end of the study protocol, animals were euthanized as per recommendation of the Panel on Euthanasia of the American Veterinary Medical Association. Accordingly, rats were placed in an empty clean chamber and the chamber was filled with 100% CO_**2**_ at a filling rate of 20–30% of chamber volume per 3–4 min. Death was confirmed by the absence of respiratory movement of the thorax. CO_**2**_ was continued for another minute after complete cessation of respiration to ensure euthanasia.

### Cardiac Magnetic Resonance Imaging (CMR)

A small animal Biospin 9.4T micro-MRI system (Bruker Biospin MRI GmbH, Ettlingen, Germany) with quadrature volume coil was used for all CMR studies. T2*-weighted and late-gadolinium (LGE) images covering the full LV were acquired. T2*-weighted images were acquired using a gradient-echo sequence (TR = R-R interval, echo time = 7.9 ms, flip angle = 8°). LGE images were acquired using an inversion-recovery prepared gradient-recalled sequence (TR/TE = 3.6/1.1 ms, flip angle = 90°) after finding the optimal time to null the remote myocardium using a scouting sequence. Spatial resolution of the MR sequences was: 200-μm in-plane resolution with 1-mm slice thickness.

### CMR analysis for infarction and iron deposition

Zone of MI and presence/extent of iron were visualized using CVI^**42**^ (Circle Cardiovascular Imaging, Calgary, AL, Canada) as previously described [17]. Briefly, endo- and epi-cardial contours were drawn to segment the left-ventricular myocardium. The remote myocardium identified on LGE images showing no hyperintensity was used as a reference region-of-interest (ROI) on both LGE and T2* images. Zone of infarction was defined as the hyperintense region on LGE images with mean signal intensity > 5 standard deviations (SD) above that of the reference regions of interest. Presence of iron within MI was identified as infarcted territory containing hypointense values that are < 2SD of the reference ROI in T2*-weighted images.

### Tissue collection

Myocardial tissue were collected from euthanized animals. The sections positive for infarction and with and without iron on the basis of CMR data were used for histopathology (tissue damage (H &E) iron deposition staining (Perl’s stain), as described above. Sham animal hearts underwent CMR to rule out infarction and representative myocardial sections were studied with H&E and Perl’s stain. Myocardial sections from animals positive for infarction also underwent immunohistochemistry. Those positive for iron on T2* and Perl’s stain within the MI zone also underwent transmission electron microscopy analysis (details below).

### Immunohistochemistry

Rats were perfused with 10 mM phosphate-buffered saline (PBS) (pH 7.4). Subsequently, the perfusion fluid was replaced by 4% phosphate-buffered paraformaldehyde (4% PFA). The heart was excised and subsequently fixed in 4% PFA for 24 hours following which the tissue was cryopreserved in 4% PFA containing 30% sucrose at 4°C. Cryostat sections (40μm) were obtained and washed (three times) in 10 mM PBS (pH 7.4). Non-specific protein interaction was blocked by incubating sections in rabbit serum prepared in 10mM PBS (with 0.01% Triton X-100) for 2 hours. Sections were washed 3 times with PBS and incubated with primary antibody solution overnight at 4°C. Primary antibodies against TNF-α (ab66579), IL-1β (ab2105) and MMP-9 (ab38898) were obtained from Abcam and prepared in 10 mM PBS (with 0.03% Triton X-100 and normal serum). The sections were then incubated in a solution of secondary antibody [Goat anti-Rabbit (1:1000; Thermo Fisher Scientific, Waltham, MA, USA), 10 mM PBS, 0.03% Triton X-100 and normal serum] for 1 h in a dark enclosure at room temperature. Immuno-labeled sections were washed and mounted on gelatin-coated slides with plain anti-fade mounting medium containing 4’,6-diamidino-2-phenylindole (DAPI) (Vector Labs). The immunostained sections were imaged using an Olympus FluoView 10i microscope (Olympus America, Center Valley, PA, USA).

### Quantitative real-time PCR

Gene expression studies were performed as previously described [18]. Briefly, total RNA was isolated from heart tissues using Trizol reagent (Sigma Aldrich, CA). The concentrations of RNA were determined using a NanoDrop spectrophotometer from the absorbance at 260nm, and RNA quality was assured with a 260/280 ratio greater than 1.8. Equal quantities of RNA were used to prepare cDNA using iScript cDNA synthesis kit (Bio-rad, CA). Real-time PCR was performed using iTaq SYBR Green Super mix with ROX (Bio-rad, CA) and gene expression was detected using the ABI Prism 7900 sequence detection system (Applied Biosystems, Foster City, CA). The primer sequences used were TNF-α (*forward*: *gtcgtagcaaaccaccaagc*, *reverse*: *tgtgggtgaggagcacatag*), IL-1β (*forward*: *gcaatggtcgggacatagtt*, *reverse*: *agacctgacttggcagaga*), MMP-9 (*forward*: *aagatgctgctgttcagcggg*, *reverse*: *gtcctcagggcactgcaggat*) and 18s mRNA (*forward*: *ttcggaactgaggccatgatt*, *reverse*: *tttcgctctggtccgtcttg*). Relative expression of genes was analyzed using the ΔΔ-Ct method and normalized to 18s mRNA levels.

### Transmission electron microscopy

Ultrastructural examination of myocardial tissue preparations was performed using electron microscopy as described previously [19]. Briefly, myocardial tissue sections were immediately fixed in a solution of glutaraldehyde (2.5%). Sections were then fixed in a solution of osmium tetroxide (1%) and embedded in Epon-Araldite. Ultrathin slices (0.1 μm thick) were obtained, stained with uranyl acetate and lead citrate, and examined in a Jeol JEM-1011 electron microscope (JEOL, Pleasanton, USA) at 80 kV, accelerating voltage and film magnification.

### Statistical analysis

Results are expressed as mean ± standard error of the mean (SEM). Statistical evaluation of the data was performed using GraphPad Prism. Data were analyzed using ANOVA, followed by Bonferroni post-hoc test wherever applicable. P value less than 0.05 was considered statistically significant.

## Results

### Late reperfusion leads to the development of hemorrhage within MI in rats

**Fig 1:**
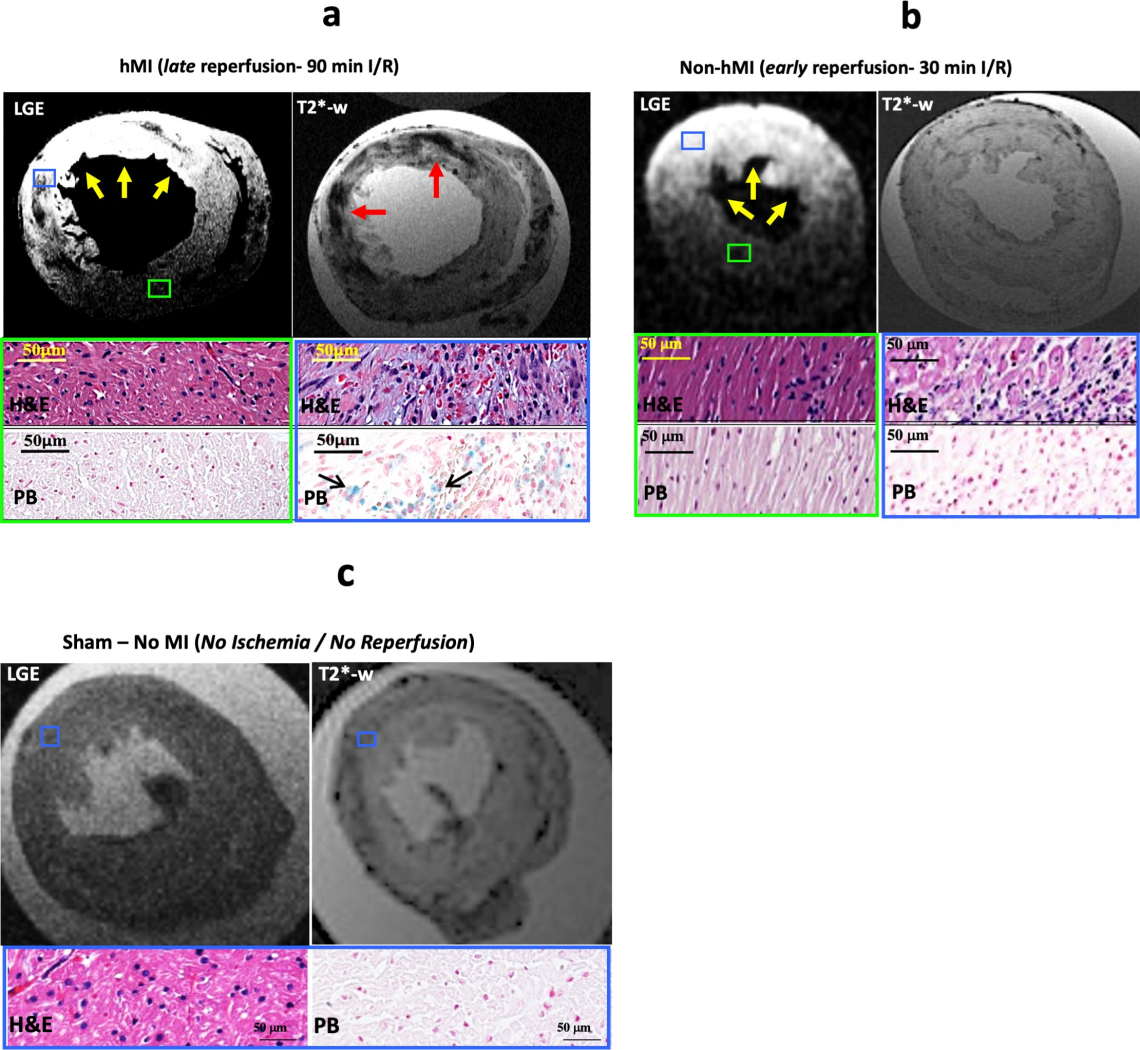
Late reperfusion leads to development of hemorrhagic MI in rats. Fig 1a-1c: Representative LGE and T2*-weighted images from ex-vivo cardiac MRI showing myocardial infarction (yellow arrows) and hemorrhage (red arrows) respectively from late-reperfusion (90-min I/R, panel a), early-reperfusion (30-min I/R, panel b) and sham (panel c) groups. Histological images with H&E staining from regions negative for MI (green box) and H&E and Perl’s stained images from regions positive for MI (blue box) are shown in late- and early-reperfusion groups. In the sham group, blue box represents region closer to the anterior descending wall.

All animals that underwent LAD ligation and reperfusion surgery survived. Representative T2*-weighted and LGE images obtained from ex-vivo hearts are shown in Fig 1. All animals with 90-min I/R were positive for hemorrhagic MI and all with 30-min I/R were not hemorrhagic. Therefore, we used the early-reperfusion (30-min I/R) and late reperfusion (90-min I/R) as models to evaluate the differential tissue specific changes in hemorrhage from non-hemorrhagic MI.

Hemorrhagic MI developed in rats subjected to late reperfusion were evidenced by LGE (yellow arrows show MI) and T2* MRI (Fig 1A). T2*-weighted MRI in late reperfusion groups showed iron deposition (red arrows) within the zone of MI as hypointensities. Histological results (blue box) verified extravasation of RBCs as an indicator of hemorrhage (H&E staining) and iron deposition (Perl’s staining) in animals that received late reperfusion. However, remote myocardial regions negative for MI and hemorrhage (green box) did not show tissue damage.

In contrast, rats subjected to early reperfusion (30-min I/R) did not show signs of hemorrhage (absence of hypointensity on T2*) with in LGE positive zones (yellow arrows) in cardiac MRI (Fig 1B). H&E stains of the myocardium (blue box) from these animals showed tissue damage but no evidence of RBC extravasation was found. Further, no stained regions positive for iron were observed within MI with Perl’s staining. Remote myocardia (green box) showed no evidence of hemorrhage or tissue damage. Sham (no ischemia/no reperfusion) rats also showed no evidence of MI or hemorrhage (Fig 1C). The quantified ex-vivo MRI measurements from all experimental groups are shown in Table 1.

**Table 1:** Late reperfusion leads to development of hemorrhagic MI in rats.

	(30-min I/R)	(90-min I/R)	Sham	Significance (vs 30-min I/R)
**Infarct Size (%LV)**	**21±6%**	**34±8%**	**0**	**P<0.05**
**Hemorrhage volume within LGE (%LV)**	**0.4±0.2%**	**9±3%**	**0**	**P<0.05**
T2* remote (ms)	**16.2±1.4**	**15.7±1.8**	**16.1±1.2**	**P>0.05**
T2* of MI (ms)	**17.1±0.1**	**5.6±0.9**	**N/A**	**P<0.05**

Quantitative CMR data on MI size, hemorrhage volume and T2* from 30-min I/R (n = 11), 90-min I/R (n = 15) and sham (n = 5) groups. Data are presented as mean±SEM. P<0.05 indicates statistical significance compared with 30-min I/R group.

### Cardiac function is worsened in rats with hemorrhagic MI

**Fig 2:**
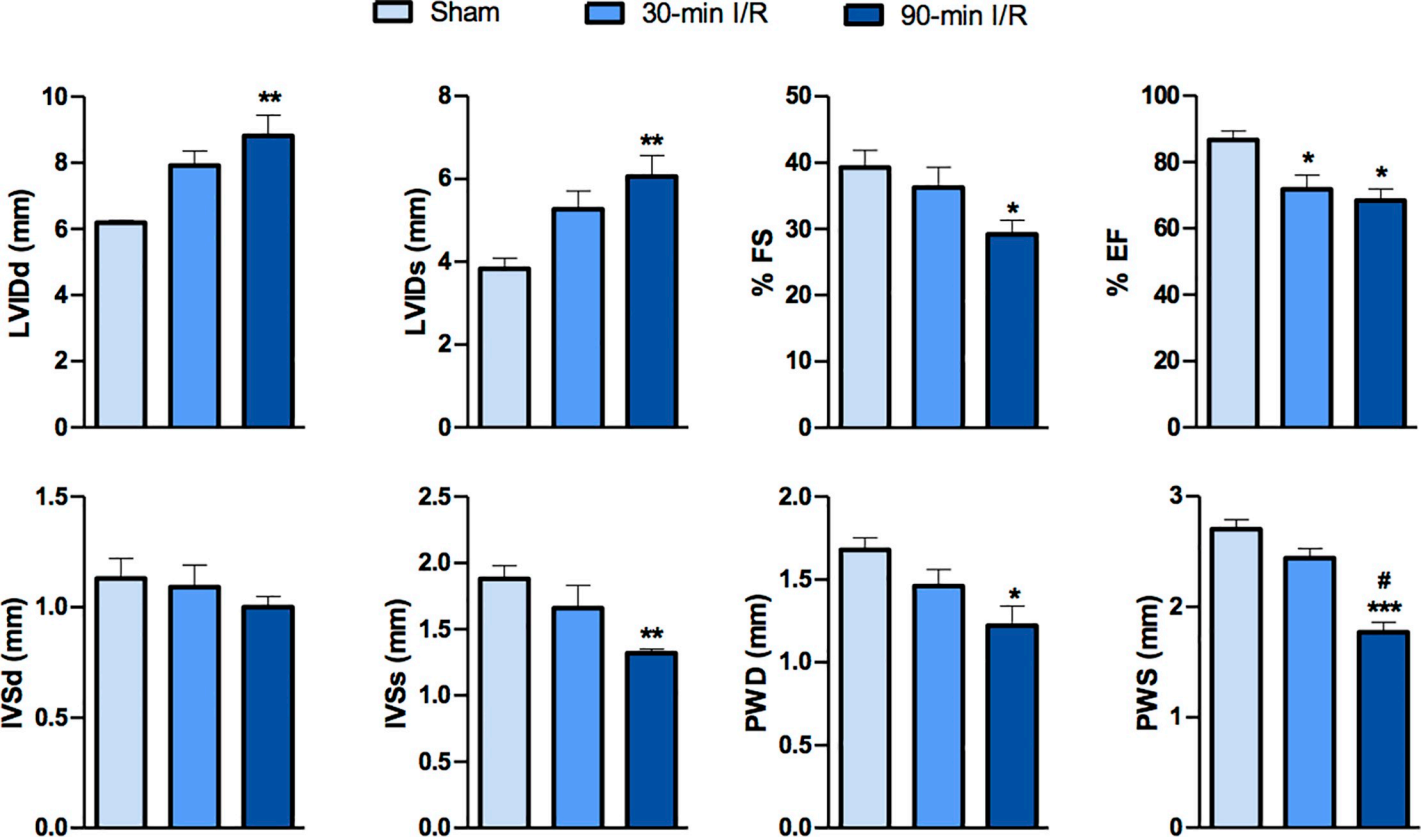
Echocardiographic parameters show adverse LV remodeling in rats with hemorrhagic MI. IVSd and IVSs (mm)—Intraventricular septal diameter at end diastole and at end systole, respectively; LVIDd and LVIDs (mm), left ventricular internal diameter at diastole and systole, respectively; PWD and PWS (mm), posterior wall thickness at end-diastole and end-systole, respectively; %FS, fractional shortening, %EF, ejection fraction (n = 7 per group). Data are presented as mean ± SEM. *P<0.05, **P<0.01 and ***P<0.001. ^#^ represents difference compared with 30-min I/R group (P<0.01).

Heart rate was not affected in any of the experimental groups irrespective of the time to reperfusion (Sham: 418±26 bpm, 30-min I/R: 431±14 bpm and 90-min I/R: 404±28 bpm, P>0.05). Fig 2 shows left-ventricular (LV) function at week 4 after I/R in rats as measured by echocardiography. Early reperfusion did not affect IVSs, PWD, PWS and fractional shortening observed in ischemic rats. However, when compared to early-reperfused rats, late-reperfusion groups failed to reverse these cardiac parameters. We observed that, in contrast to 30-min I/R, 90-min I/R groups showed decreased IVSs, PWD, PWS and %FS in comparison to sham controls. In comparison to sham animals, ejection fraction (%EF) was significantly reduced in both 30-min I/R and 90-min I/R groups. Additionally, 90-min I/R groups exhibited significantly decreased PWS, when compared to 30-min I/R group, suggesting that late-reperfusion induced hemorrhagic MI might have certain acute effects on cardiac function.

### Rats with intramyocardial hemorrhage demonstrate proinflammatory burden within chronic MI territories

**Fig 3:**
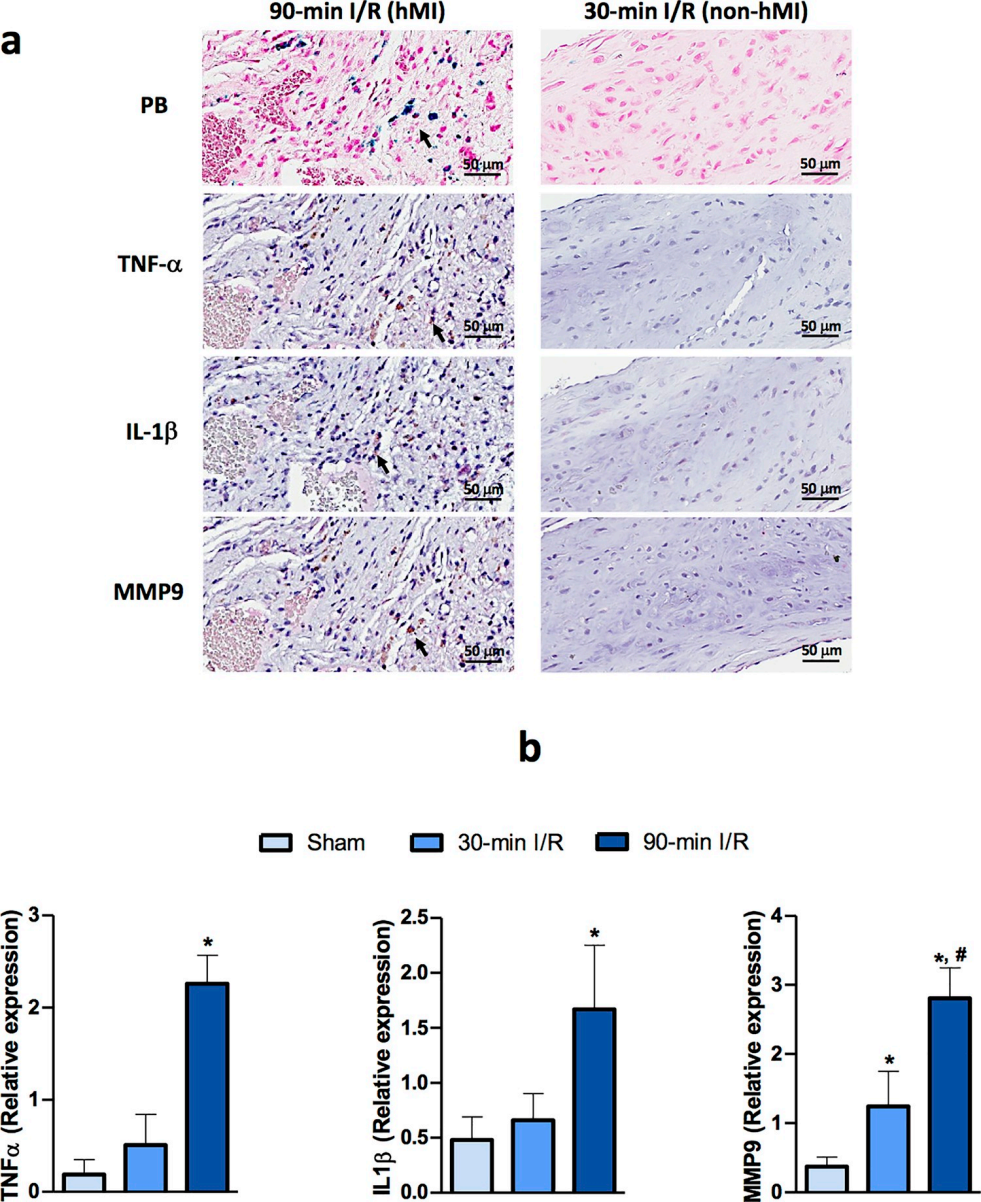
Proinflammatory burden in chronic hemorrhagic MI territories in rats. Panel a show hemorrhagic MI region with proinflammatory cytokines. Representative immunohistochemistry images of proinflammatory activation in hemorrhagic myocardial infarction are shown with immunoreactivity of inflammatory cytokines TNF-α and IL-1β, and fibrotic marker MMP-9 in myocardial tissue from 30-min I/R and 90-min I/R groups. Panel b shows gene expression of proinflammatory cytokines in rats with hemorrhagic MI. mRNA expression levels of the indicated genes in heart tissue from rats that underwent sham surgery, 30-min I/R and 90-min I/R (n = 7/group). Data are presented as mean ± SEM. *P<0.05 compared with sham, ^#^P<0.05 compared with 30-min I/R group.

Compared to animals subjected without evidence of intramyocardial hemorrhage, inflammatory signaling was upregulated in animals with hemorrhagic MI as evidenced by increased protein expression levels of the proinflammatory cytokines, IL-1β and TNF-α (Fig 3A). Expression of the fibrotic marker MMP-9 was also elevated in rats with hemorrhagic MI when compared to non-hemorrhagic MI groups. We observed significantly augmented immunoreactivity to TNF-α, IL-1β and MMP-9 in the myocardial sections of rats that had underwent delayed-reperfusion induced hemorrhagic MI.

We also investigated changes in gene expression of these proinflammatory markers between the early- and late-reperfusion groups (Fig 3B). The expression levels of TNF-α, IL-1β and MMP-9 mRNA were significantly elevated in late-reperfused hemorrhagic tissue compared to non-hemorrhagic hearts (TNF α: 2.26±0.3 vs 0.51±0.3, IL-1β: 1.67±0.6 vs 0.66±0.2 and MMP-9: 2.81±0.4 vs 1.24±0.5, n = 7, P<0.05).

### Ultrastructure of chronic hemorrhagic MI regions in rats revealed granular iron traces within phagosomes

**Fig 4:**
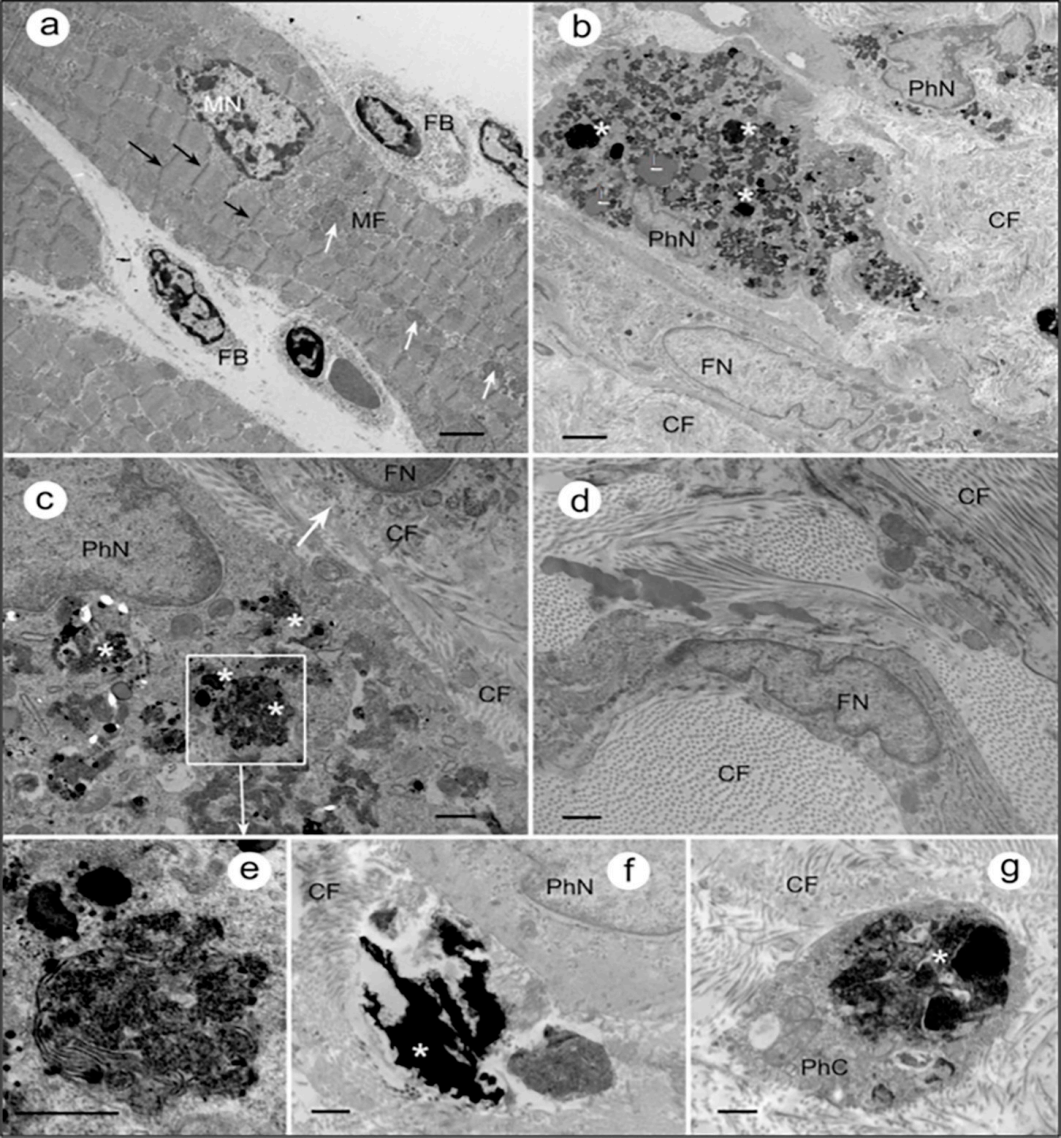
Granular traces of iron are evident in the ultrastructure of the hemorrhagic MI in rats. a) Representative TEM image of a section through the myocardium of sham control rat. Black arrows indicate z-discs; white arrows show mitochondria. FB–fibroblast; MN–myocyte nucleus. Scale bar: 2um b) Section through the myocardium of rat with delayed reperfusion that was confirmed for iron within MI in T2* CMR and Perl’s staining. Organization of myocardial tissue is chaotic, no traces of regularly arranged myofibrils can be observed. Instead, the tissue is infiltrated with large fibroblast actively producing collagen fibers (CF). Accumulations of granular material within the phagosomes (white asterisks), are consistent with expected appearance of aggregated iron particles. FN, fibroblast nucleus, PhN, phagocyte nucleus. Scale bar: 2μm c) Section through another region of the myocardium that underwent delayed reperfusion post MI: a phagocyte with numerous phagosomes/secondary lysosomes loaded with aggregates of iron particles (asterisks), and a fibroblast (arrow) with intracellular and extracellular collagen fibers are in the view. Scale bar: 0.5μm d) Fibroblasts and collagen fibers filling the myocardial tissue after delayed-reperfusion. Scale bar: 0.5μm e) Fragment of panel (c) at higher magnification: phagosomes with iron particles inside (asterisk). Scale bar: 0.5μm f) Accumulation of iron (asterisk) outside the phagocytic cell. PhN, phagocyte nucleus. Scale bar: 0.5μm g) A phagosome with iron aggregates (asterisk) in the phagocyte cytoplasm (PhC) surrounded by CF. Scale bar: 0.5μm.

Fig 4 shows transmission electron micrographs revealing ultrastructural features of myocardium from rats with sham operation and hemorrhagic MI. In the sham group, myocytes demonstrate highly organized internal structure with regularly arranged myofibrils (MF). In contrast, myocardium from rats subjected to late reperfusion showed no traces of regularly arranges myofibrils. Instead the tissue was observed to be infiltrated with large fibroblasts actively producing collagen fibers. Further, consistent with our T2* imaging data (Fig 1), accumulation of granular aggregated iron particles was also observed within the phagosomes from myocardium of late reperfusion rats. No traces of iron accumulation were evident within the organelles from sham myocardium.

## Discussion

Patients with hemorrhagic MIs have an increased risk for major adverse cardiovascular events (MACE) [7, 20, 21]. However, long term consequences of hemorrhagic MIs are not completely understood. Importantly, the mechanism by which hemorrhage drives cardiovascular morbidity is unclear. One of the major hindrances in studying the adverse effects associated with hemorrhagic MI is the lack of availability of cost-effective animal models for hemorrhagic MI. Herein, we investigated the physiological conditions inducing hemorrhagic MI in a rodent model, which can evolve as a formidable tool for conducting mechanistic studies.

We found that delayed reperfusion induces hemorrhagic MI in rats and is characterized by elevated iron remnants within the infarct zones in the chronic phase of the infarction. Although multiple studies have used cardiac MRI to detect the acute effects of intramyocardial hemorrhage in rat models, the objective of these studies were not centered on establishing a rat model for hMI to investigate consequences associated with hMI. A study by Ye et al, which examined reperfused MIs (120 mins of LAD occlusion followed by reperfusion) in rats showed that all 28 rats in the study, except one, was hemorrhagic. Taken together with our findings here that a 90-min I/R always induces hMI, our findings are to a large extent consistent with that of Ye et al, except for the 1 rat (out of 28) in their study not developing hMI likely to have been an outlier [22, 23]. We speculate that this minor discrepancy could also be due to a variation in the exact location along the LAD where the occlusion was introduced, or a result of the specific rodent strain used (wistar vs sprague dawley). Moreover, our studies on the contrary investigated ischemia time as a key component of when hemorrhagic MIs can be expected so that we can propose experimental criteria driving intramyocardial hemorrhage in rats. We also used T2*-weighted CMR to detect intramyocardial hemorrhage in ex-vivo rodent hearts in the acute phase and chronic iron deposits within the infarcted territories. This is consistent with our previous findings in large animal models and in patients, which forms additional support for the use of the rat model for investigating physiological consequences of hemorrhagic MIs [9, 10]. Further, Perl’s staining confirmed accumulation of iron within the infarcted areas in rats that received late reperfusion. Our data from H&E stained sections supports the notion that hemorrhage in these animals was likely caused by the extravasation of RBCs. Interestingly, rats subjected to early reperfusion showed tissue damage but there was no evidence for red blood cell extravasation (hemorrhage) in the acute phase or iron deposition in the chronic phase of MI, further confirming that there are key differences between hemorrhagic and non-hemorrhagic MIs.

Previous studies have associated MI with cardiac remodeling and a decline in cardiac function [7, 24, 25]. Specifically, the loss of viable myocardium induced by MI has been shown to impair cardiac function by lowering LV ejection fraction and LV fractional shortening [26]. The left ventricular performance and its interrelationships between MI size and mortality-morbidity rates are well established [27, 28]. However, the nature and extent of functional impairment associated with hemorrhagic MI is not completely understood. Consistent with data in patients and large animal models, our data in rodents support the notion that hemorrhagic MI induces a further decline in left ventricular function compared to non-hemorrhagic MI. Compared to the early reperfusion group, the late reperfusion group demonstrated significant increases in LV internal diameter and marked reductions in LV fractional shortening, indicating that hemorrhage might even accelerate the progression to chronic heart failure. It is understandable that the decline in cardiac function might be an effect of the extended ischemia in delayed reperfused groups. Therefore, additional studies clarifying the consequence of extended ischemia *per se* on cardiac remodeling and functional decline is warranted.

In addition to the demonstration that hemorrhagic MIs result in iron deposition, which can be detected by T2*-weighted cardiac MRI, we extended these findings by providing evidence of granular iron accumulation within MIs based on scanning transmission electron microscopy (TEM) of myocardium from late reperfusion groups. We also showed that late reperfusion leads to disorganization of the myocardial tissue leaving no traces of regular myofibril bands. Further, our TEM images depict the presence of phagocytes containing phagosomes, suggestive of increased phagocytosis in hemorrhagic MI. Also, we showed that the accumulation of granular electron-dense material within these phagosomes, are likely iron deposits. This is indeed in line with previous findings in large animal models of hemorrhagic MI and may help elucidate the role of inflammatory activators in chronic MI with aggregate iron particles (in the form of ferric iron) [9]. Previous *in vitro* studies have shown that when macrophages and other cell types are incubated with iron, NF-κB signaling pathway is activated [29, 30]. Although, inflammatory cytokines activated by the transcription factor NF-κB, such as TNF-α and IL-1β, and their relationships with MI are well established, their critical role in the context of hemorrhagic MI are not understood [31]. Further, studies have shown that healthy cardiomyocytes that do not normally express TNF-α produce large amounts of this inflammatory cytokine once these cells are affected by ischemia or anoxia post-MI [32, 33]. Our immunostaining data in this study demonstrating increased TNF-α and IL-1β expression in rats with hemorrhagic MI indicates that elevated iron deposition in hemorrhagic MI might be driving the proinflammatory burden in myocardial cells. Furthermore, we also show that hemorrhagic MI induced an increase in expression of MMP-9, which has been previously shown to be a key instigator of LV remodeling post-MI. MMP-9, in particular, is known to coordinate multiple aspects of LV remodeling by modulating inflammatory protein turnover as well as exerting indirect effects on leukocytes and fibroblasts that are responsible for myocardial wound healing. Taken together, it is plausible that elevated iron depositions in hemorrhagic MI is a major factor contributing to the overactivation of inflammatory cascade leading to accelerated structural and functional cardiac damage.

Despite our study being the first to elucidate the conditions to consistently recapitulate the pathophysiological features of hMI, there are limitations. First, although previously established in large animal models, we do not have data to support a quantitative correlation between iron deposition and MI severity in rats at this time. However, the characteristic similarities of hemorrhagic MI observed in rats with previously reported large animal models (increased proinflammatory burden, functional and structural myocardial changes) provides support that the proposed rat animal model is robust for evaluating paralleled observations in large animals and humans. Second, we did not establish the mechanistic link between iron deposition and chronic proinflammatory protein expression. The intent of the present study was to establish a small animal model to study molecular aspects of hemorrhagic MI. Accordingly, future studies using this model is expected to enable investigations into the role of iron deposition following hemorrhagic MI in propagating inflammatory burden within the myocardial tissue and to investigate mechanistic pathways driving adverse outcomes in hemorrhagic MI. Third, we were not able to perform in-vivo CMR. The in-vivo studies were performed at one site (LSU), which did not have access to appropriate MRI equipment and transfer of live animals with MI was not possible to an alternate site with MRI equipment due to institutional requirements. To mitigate this limitation, we chose to conduct ex-vivo studies at the second collaboration site (Cedars-Sinai Medical Center). While it would have been ideal to perform in-vivo studies, ex-vivo CMR studies provide a pathway for establishment of rat model of hMI. However, the relative differences between ex-vivo CMR evidence for hMI remains to be validated in in-vivo CMR. Finally, we only investigated incidence of hemorrhage in male rats. A separate study is currently underway investigating the relationship between sex and the incidence of hemorrhage in reperfused MI.

In summary, we have demonstrated specific conditions that would forge the development of hemorrhagic MI in rats, which could serve as a critical tool to study the underlying mechanisms driving differential outcomes in hemorrhagic versus non-hemorrhagic MI. Our findings also show that the hemorrhagic MIs in rats mirror clinical and large animal evidence of chronic iron deposition. This support the notion that the proposed rat model is capable of providing a meaningful starting point to investigate the mechanistic aspects of hemorrhagic MIs with findings that could lend translational outlook. Further the proposed model could help refine myocardial research through emphasis that not all myocardial infarctions should be treated equal.
